# Commentary: Gut dysbiosis in patients with chronic pain: a systematic review and meta-analysis

**DOI:** 10.3389/fimmu.2024.1445334

**Published:** 2024-09-26

**Authors:** Takahiko Nagamine

**Affiliations:** Department of Psychiatric Internal Medicine, Sunlight Brain Research Center, Hofu, Japan

**Keywords:** chronic pain, dopamine, dysbiosis, estrogen, gut brain axis

## Introduction

Chronic pain has been demonstrated to significantly reduce an individual’s quality of life. Various treatment modalities have been employed to address this condition, including pharmacotherapy, cognitive-behavioral therapy, and transcranial magnetic stimulation therapy (1). However, there is currently no established treatment for chronic pain due to the incomplete understanding of its pathogenesis. It has recently been proposed that the gut microbiota may play a role in the etiology of these conditions (2). I found the article by Goudman et al. on the meta-analysis of the gut microbiota in patients with chronic pain to be of great interest. The study demonstrated that the gut microbiota of individuals with chronic pain exhibited dysbiosis, which resulted in a reduction in alpha diversity and a relative increase in the genus *Eggerthella* (3). The reduction in alpha diversity is associated with a decline in bacteria that produce short-chain fatty acids, such as butyrate (4). This weakens the barrier function of the intestinal epithelium, making it easier for information about changes in the intestinal environment, such as an increase in *Eggerthella* spp., to be transmitted directly to the brain. The purpose of this paper is to examine the impact of *Eggerthella* spp. on the development of chronic pain.

## Chronic pain and dopamine

The genus *Eggerthella* are involved in dopamine metabolism. For instance, *Eggerthella lenta* has the capacity to dehydroxylate dopamine (5). In the intestines of individuals diagnosed with Parkinson’s disease, this bacterium has been demonstrated to degrade dopamine to m-tyramine (5). It is important to note that dopamine does not cross the blood-brain barrier, and therefore, dopamine in the digestive tract does not migrate to the central nervous system. Nevertheless, the observed increase in genera containing bacteria that affect dopamine metabolism indicates that dopamine metabolism is altered in the central nervous system. This is likely due to the fact that the brain and gut are connected via the brain-gut axis. However, it is currently unclear whether this phenomenon is a cause or an effect. Dopamine in the central nervous system exerts an analgesic effect by stimulating dopamine D2 receptors in the dorsal striatum and nucleus accumbens during persistent pain. Increased dopaminergic output from the striatal dopamine loop stimulates the reward system and the periaqueductal gray, the origin of the descending pain inhibition pathway (6). Consequently, the diminished activity of striatal D2 receptors and diminished output of the striatal dopamine loop are associated with chronic pain (7). Chronic pain is associated with decreased dopamine function, which renders it susceptible to coexistence with other conditions, such as depression and Parkinson’s disease, which are also associated with decreased dopaminergic neurotransmission (8). In addition, reduced function of the dopamine loop in the basal ganglia promotes pain at the level of the spinal cord. Parkinson’s model mice in which dopamine cells were reduced by administration of a small amount of 6-hydroxydopamine to the midbrain exhibited chronic pain, the mechanism of which is spontaneous excitatory postsynaptic currents in the superficial dorsal horn. The reduced pain threshold of these Parkinson’s model mice was improved by stimulating the dopamine D2 receptor with a dopamine agonist (9).

**Figure 1:**
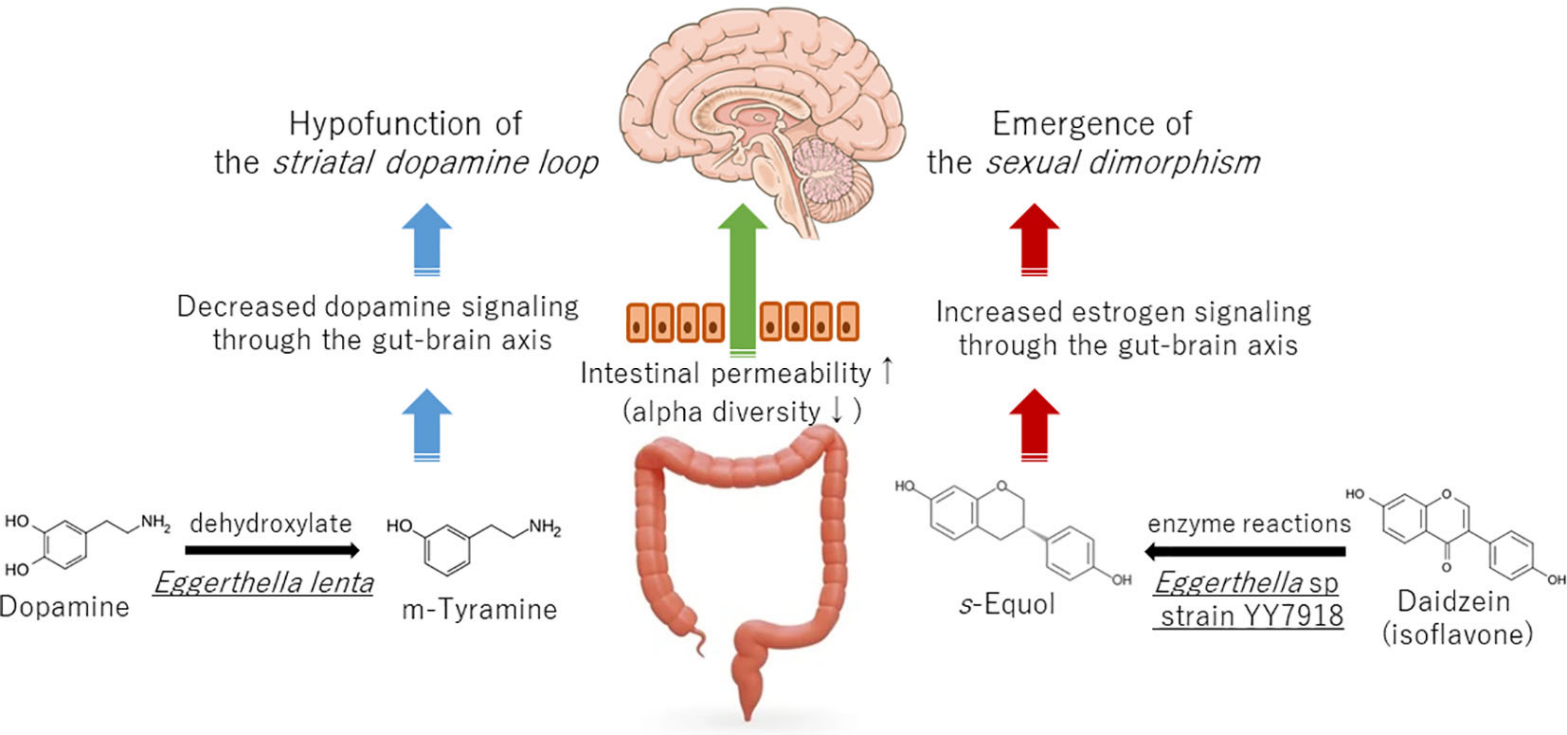
Effect of *Eggerthella* spp. on the development of chronic pain via dopamine and estrogen signaling.

Nevertheless, pharmacotherapy for chronic pain that regulates monoamines primarily targets serotonin, as evidenced by the administration of antidepressants (10). This is because serotonin is the primary neurotransmitter in the descending pain inhibitory pathway. Research on the gut microbiota of chronic pain patients has focused on tryptophan-producing bacteria. It has been demonstrated that excessive activation of microglial cells, which use tryptophan as a raw material, is caused by a decrease in serotonin and melatonin in the brain. This leads to increased pain and the coexistence of depression and insomnia (11). However, treatment with tricyclic antidepressants for chronic pain can be efficacious in certain instances but less so in others (12). Moreover, drugs that only enhance serotonin, such as selective serotonin reuptake inhibitors, can cause chronic pain, as is known as the serotonin paradox (13). In light of the findings by Goudman et al. indicating a potential association between gut microbiota characteristics and central dopamine dysfunction in chronic pain patients, it is recommended that the benefits of combination therapies, which aim to maintain the function of the striatal dopamine loop, be considered alongside conventional serotonin-targeting therapies in the pharmacological treatment of chronic pain (**Figure 1**).

## Chronic pain and estrogen

The genus *Eggerthella* may also be associated with the regulation of female hormones. For instance, *Eggerthella* sp. YY7918, which is found in human feces, metabolizes isoflavonoids to produce S-equol, which has been demonstrated to possess strong estrogenic activity (14). The majority of chronic pain disorders are more prevalent in women than in men, with sex ratios varying considerably by disease (15). For instance, the prevalence of the burning mouth syndrome, a chronic orofacial pain, is three to five times higher in women than in men, with a higher incidence observed in peri- and postmenopausal women. One potential explanation for the heightened prevalence of chronic pain among menopausal women is the involvement of estrogen in the pathogenesis of chronic pain at two distinct stages: during puberty and menopause. This phenomenon is known as the two-hit theory by estrogen (16). The expression of the transient receptor potential vanilloid 1 (TRPV1), a pain receptor, is increased by estrogenic effects and is associated with chronic facial pain (17). As a first hit by estrogen, increased estrogen during puberty increases TRPV1 expression, making at-risk individuals more sensitive to pain. On the other hand, estrogen alleviates pain by downregulating nerve growth factor (NGF), which can translocate TRPV1 to the cell surface membrane. As a second hit by estrogen, when estrogen decreases during menopause, NGF increases, TRPV1 increases at the cell surface, pain sensitivity increases, neuroinflammation also occurs, and chronic pain develops in at-risk individuals (16). In fact, TRPV1 expression is increased in the oral mucosa of menopause chronic orofacial pain patients (18). First of all, the intestinal flora is influenced by estrogen, and its composition differs according to sex, known as the “estrobolome”. During puberty, estrogen induces a female-type intestinal flora (19), and during menopause, the intestinal flora becomes more similar to that of male (20). Both of these stages of change may be involved in the expression of pain.

Moreover, studies employing animal models have demonstrated that the expression of monoamine receptors in the descending pain inhibitory pathway is influenced by estradiol administration (21). It is evident that sexual dimorphism exists in various components of the pain circuitry such as the descending pain inhibitory pathway and the bed nucleus of the stria terminalis (22). The gut microbiota of chronic pain patients may transmit estrogen stimulation to the brain, and one of these may be *Eggerthella* sp. (**Figure 1**).

## Conclusion

In conclusion, further investigation is required to determine whether the gut microbiota of chronic pain patients reduces central dopamine function or has an estrogen-like stimulating effect. The gut microbiota may represent a potential therapeutic target for chronic pain patients. Probiotics that produce short-chain fatty acids including butyrate have been demonstrated to be efficacious in restoring dopamine function in Parkinson’s disease (23). Therefore, it would be beneficial to investigate whether probiotics that produce short-chain fatty acids can also improve chronic pain. The mounting evidence indicating the pivotal role of the gut microbiota in regulating chronic pain has opened up new avenues in the field of pain management.
